# Neurofunctional and Clinical Effects of Intranasal Human Recombinant Nerve Growth Factor in Children with Acquired Brain Injury

**DOI:** 10.3390/ph19040590

**Published:** 2026-04-07

**Authors:** Lorenzo Di Sarno, Serena Ferretti, Lavinia Capossela, Antonio Gatto, Valeria Pansini, Luigi Manni, Antonio Chiaretti

**Affiliations:** 1 Dipartimento di Pediatria, Fondazione Policlinico Universitario “A. Gemelli” IRCCS, 00168 Rome, Italyantonio.gatto@policlinicogemelli.it (A.G.);; 2 Istituto di Farmacologia Traslazionale, Consiglio Nazionale delle Ricerche (CNR), 00133 Rome, Italy; 3 Dipartimento di Pediatria, Università Cattolica del Sacro Cuore, 00168 Rome, Italy; 4 Department of Women’s Health Sciences, Fondazione Policlinico Universitario “A. Gemelli”IRCCS, Largo Agostino Gemelli 8, 00168 Rome, Italy

**Keywords:** human-recombinant nerve growth factor, intranasal administration, meningitis, cardiac arrest, neuroprotection, transcranial direct current stimulations, traumatic brain injury

## Abstract

**Background**: Traumatic brain injury (TBI) and hypoxic-ischemic encephalopathy (HIE) cause significant pediatric morbidity through primary insults and secondary cascades like excitotoxicity, neuroinflammation, and impaired plasticity. Nerve growth factor (NGF) promotes neuroprotection, anti-inflammation, and repair, but delivery challenges persist. This review evaluates preclinical and clinical evidence on intranasal human recombinant NGF (hr-NGF) to enhance neurorepair in pediatric TBI and HIE patients. It aims to clarify the potential of intranasal hr-NGF as part of future multimodal approaches to enhance brain repair and improve functional recovery across the lifespan. **Methods**: A PRISMA-guided literature search (2000–2025) was conducted across Scopus, PubMed, and Cochrane CENTRAL using terms like “intranasal NGF”, “TBI”, “HIE”, and “pediatric”. Eligible studies involved pediatric brain injury patients receiving NGF, with outcomes via clinical scales, imaging, or EEG. **Results**: Preclinical models showed that intranasal NGF reduces lesion volume, inflammation, and deficits while boosting angiogenesis and cholinergic function. Clinically, one child with meningitis and five TBI cases exhibited improved consciousness, spasticity, motor scores, cognition, and brain imaging. Three HIE cases gained voluntary movements, expressivity, and perfusion. No adverse events occurred related to hr-NGF administration. **Conclusions**: Intranasal hr-NGF safely reactivates plasticity in pediatric brain injury, yielding motor, cognitive, and neurophysiological gains. Preliminary data support multimodal use, but randomized trials are needed to optimize protocols and confirm efficacy.

## 1. Introduction

Traumatic brain injury (TBI) is a major cause of death and long-term disability worldwide in children [1,2]. It involves an immediate mechanical insult followed by secondary injury mechanisms, including neuroinflammation, excitotoxicity, oxidative stress, mitochondrial dysfunction, and blood–brain barrier disruption, which together exacerbate neuronal damage and impair endogenous repair processes [3]. As a result, many survivors develop persistent cognitive, behavioral, and motor deficits. Despite advances in critical care, effective pharmacological and non pharmacological therapies that reliably restore neuronal integrity after TBI are still lacking [4].

Similarly, hypoxic-ischemic encephalopathy (HIE) is a leading cause of acquired brain injury in neonates, resulting from perinatal asphyxia and reduced cerebral perfusion [5]. Moderate to severe HIE carries a high risk of death or permanent neurological disability, including cerebral palsy, epilepsy, cognitive impairment and sensory deficits [5]. Although therapeutic hypothermia improves survival and outcomes, its efficacy is partial and strongly time-dependent, leaving many infants with significant residual deficits [6]. Several adjunctive neuroprotective strategies, such as erythropoietin, stem cell-based therapies, xenon, and melatonin are under investigation, but none has yet achieved sufficient evidence for routine clinical use [6].

In this context, neurotrophic factors have gained attention as potential modulators of brain repair. Nerve growth factor (NGF) is a central regulator of neuronal survival, differentiation, and synaptic plasticity [7]. Experimental models of neurotrauma and neurodegeneration show that NGF can attenuate neuroinflammation, limit apoptosis, and promote structural and functional recovery after brain injury [8], making it an attractive candidate for both traumatic and hypoxic-ischemic conditions. Specifically, NGF exerts its neuroprotective effects by activating the high-affinity TrkA receptor, which triggers downstream PI3K/Akt and MAPK/ERK signaling pathways to counteract the pro-apoptotic cascades and oxidative stress characteristic of TBI and HIE pathophysiology.

However, clinical translation is limited by difficulties in delivering NGF to the brain safely and effectively [9]. While intracerebral or intraventricular administration bypasses the blood–brain barrier, it requires invasive neurosurgical procedures and carries significant risks [10]. Intranasal delivery has emerged as a non-invasive alternative that exploits olfactory and trigeminal pathways to transport NGF directly to the central nervous system [9]. Preclinical and early clinical data suggest that intranasal NGF can achieve therapeutically relevant brain concentrations, supporting its feasibility for repeated or chronic use [11]. While eye drops are specifically intended for localized ocular pathologies, the intranasal route is superior for bypassing the blood–brain barrier and achieving effective penetration to reach damaged brain areas.

This review will therefore explore the role of NGF in pediatric acquired brain injury, focusing on TBI and HIE as paradigmatic models. The decision to focus on pediatric patients is inevitable, as there are currently no studies in the literature on adult patients with the same conditions treated with intranasal NGF. This review will summarize NGF-related mechanisms, evaluate available preclinical and clinical evidence, and discuss key translational challenges, including timing, dosing, and delivery strategies. By integrating experimental and clinical insights, it aims to clarify the potential of intranasal NGF as part of future multimodal approaches to enhance brain repair and improve functional recovery across the lifespan, trusting that the preliminary data currently available on a limited number of patients can be confirmed in the future with large-scale trials. This paper represents a comprehensive synthesis of the authors’ collective clinical and research experience, integrating longitudinal observations with current theoretical frameworks.

## 2. Materials and Methods

We performed a literature search to identify studies evaluating intranasal hr-NGF in pediatric brain injury and reports published between 1st January 2000 and 1st December 2025; the following electronic databases were systematically searched: Scopus; PubMed; and Cochrane Central Register of Controlled Trials (CENTRAL). The search was performed according to the Preferred Reporting Items for Systematic Reviews and Meta-Analysis (PRISMA) method [12].

The research strings were as follows:

“intranasal nerve growth factor” OR “intranasal NGF” OR “nerve growth factor” OR “NGF” OR “recombinant human nerve growth factor” OR “rhNGF” OR “neurotrophin” OR “neurotrophic factor” OR “neuroplasticity” OR “brain plasticity”

AND

“traumatic brain injury” OR “TBI” OR “brain injury” OR “acquired brain injury” OR “hypoxic-ischemic encephalopathy” OR “hypoxic ischemic encephalopathy” OR “HIE” OR “perinatal hypoxic ischemic encephalopathy” OR “neonatal hypoxic ischemic encephalopathy” OR “hypoxic brain injury”

AND

“child” OR “pediatric” OR “paediatric” OR “infant” OR “neonate” OR “newborn”.

Studies were deemed eligible for inclusion if they met the following criteria: (1) enrollment of pediatric patients diagnosed with brain injury; (2) administration of intranasal NGF as part of the therapeutic intervention; and (3) assessment of neurological outcomes using standardized clinical scales—such as the Glasgow Coma Scale (GCS), the Coma Recovery Scale–Revised (CRS-R), or similar instruments—or through radiological, biochemical, or electroencephalographic (EEG) evaluations. Exclusion criteria comprised publications not written in English and studies in which neurological outcomes were neither quantified using validated assessment scales nor evaluated by radiological investigations or EEG recordings. The present review primarily focused on retrospective observational studies or prospective interventional studies and preclinical models. Study selection was conducted independently by two reviewers (LDS and SF). All relevant articles identified during the initial screening were subsequently examined in detail to retrieve additional references not captured in the preliminary search. Review articles and commentaries lacking original data were excluded; however, their content was consulted to aid interpretation and contextualization of the collected evidence.

A comprehensive appraisal of methodological quality was performed, with particular attention to the following sources of bias: baseline comparability of participants with respect to key prognostic factors (homogeneity bias); concealment of allocation (selection bias); blinding of outcome assessment (detection bias); completeness of outcome data (attrition bias); and avoidance of concomitant interventions (co-intervention bias). It should be acknowledged that case reports and case series are intrinsically susceptible to bias because of their uncontrolled nature, absence of randomization, and vulnerability to selection and information biases, thereby limiting their suitability for drawing definitive conclusions regarding efficacy. Nonetheless, such study designs remain valuable for hypothesis generation, as they may uncover novel findings and potential therapeutic effects that inform and stimulate future research.

## 3. Results

**Figure 1:**
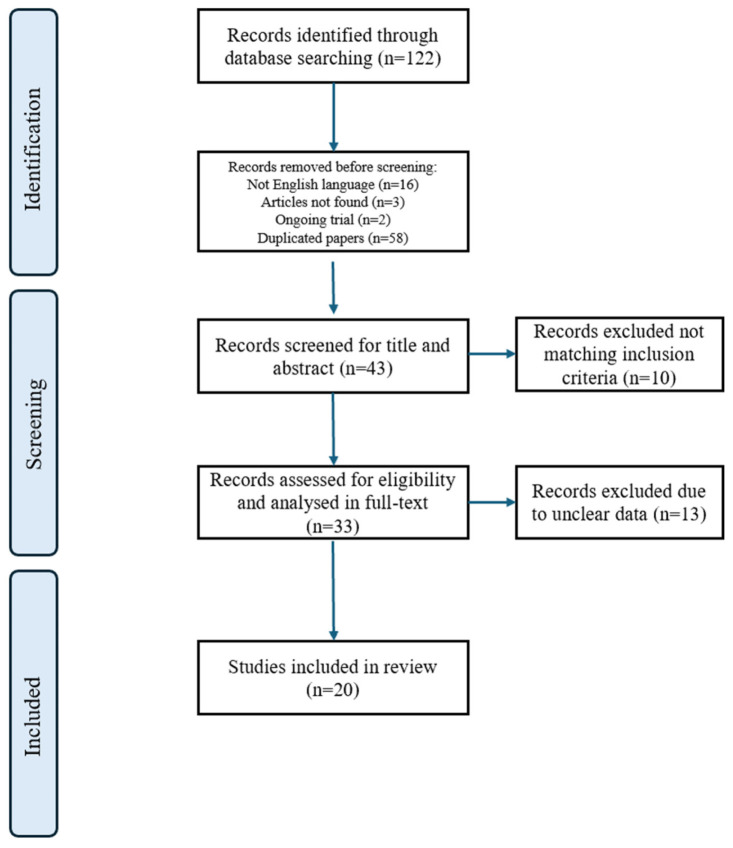
Flow diagram of literature selection.

Overall, we identified 122 records through database searching. As a first step, we excluded 16 articles in a non-English language, 3 records whose related articles were not available, 2 articles concerning ongoing trials, and 58 duplicated papers. As a second step, we eliminated 10 records by evaluating only title and abstract because they did not match the inclusion criteria above-mentioned. Of the remaining 33 studies, we excluded 13 through a further discussion among the authors on the reliability of the data. These studies—2 clinical and 11 preclinical—were excluded (by LDS, SF and LM) mainly due to the presence of confounding variables and selection bias. Thus, 20 selected articles were included in the review. The detailed selection of the literature is shown in Figure 1.

TBI and HIE involve an initial mechanical or ischemic insult followed by secondary cascades of excitotoxicity, neuroinflammation, oxidative stress, blood–brain barrier disruption, and neuronal apoptosis, which ultimately lead to persistent cognitive, motor, and behavioral impairments [13,14]. Preclinical rodent models, such as controlled cortical impact (CCI), fluid percussion injury (FPI), and the Rice–Vannucci model of neonatal HIE, faithfully reproduce these pathological processes and have highlighted the broad neuroprotective and neurorestorative actions of NGF [15]. Although originally characterized in the peripheral nervous system, NGF has therefore emerged as a key regulator of central nervous system (CNS) plasticity and repair in experimental brain injury.

### 3.1. Preclinical Insights

Experimental models show that the adult brain can upregulate NGF as an intrinsic response to injury. After cortical trauma in rats, NGF protein and mRNA rapidly increase in the cortex and hippocampus [16,17], mainly in reactive astrocytes rather than neurons, as confirmed by dual-labeling immunocytochemistry combining in situ hybridization with glial fibrillary acidic protein (GFAP) immunostaining, indicating a glial-driven neuroprotective attempt [18]. This response is regulated by inflammatory signaling, as IL-1β blockade significantly reduces NGF upregulation [16]. Similar findings are observed after hypoxic injury, where NGF levels rise in the neostriatum, hippocampus, and neocortex, correlating with focal neuronal degeneration and inflammation and reflecting enhanced synthesis by activated glial and immune cells [19].

However, therapeutic hypothermia, while reducing edema and inflammation, suppresses IL-1β signaling and abolishes endogenous NGF upregulation in TBI rat models [20], suggesting that standard neuroprotection may inadvertently inhibit intrinsic repair pathways. After ischemic injury, NGF expression follows a biphasic pattern, with an early decrease and a later compensatory increase [2], indicating that the acute phase may represent a window of NGF deficiency amenable to early supplementation [2,15].

Exogenous NGF administration produces robust functional and structural benefits. In TBI rats, chronic intraventricular NGF infusion improves cognitive performance and preserves spatial memory [21], protects cholinergic neurotransmission, restores hippocampal acetylcholine release, and prevents the degeneration of choline acetyltransferase (ChAT)-positive neurons in the medial septum [21,22]. In ischemic and hypoxic models (rats), NGF reduces infarct volume and improves neurological outcomes [8], with effects extending beyond classical cholinergic targets. These benefits are partly mediated by angiogenesis through Vascular Endothelial Growth Factor (VEGF) induction and activation of the TrkA, PI3K/Akt, and ERK1/2 pathways [23], potentially involving HIF-1α under hypoxic conditions [24]. NGF also supports oligodendrocyte precursor survival and maturation, contributing to white matter preservation and remyelination [25].

Recent studies emphasize the feasibility of non-invasive NGF delivery. Intranasal administration effectively transports NGF to the brain, reduces neuroinflammation, shifts microglia toward a neuroprotective phenotype, and limits Tau and amyloid-β accumulation after TBI [9,11,15]. Acute intranasal NGF in young TBI rats reduces lesion volume and neuronal loss, induces protective astrocytic phenotypes, lowers pro-inflammatory cytokines such as IL-1β, and enhances angiogenic responses [11]. In neonatal HIE, intranasal administration of the modified “painless” NGF (CHF6467) significantly reduces infarct size and shows synergistic neuroprotection when combined with hypothermia, improving motor coordination and long-term memory while decreasing neuroinflammatory and neuroaxonal damage markers [26].

Overall, preclinical evidence supports a multimodal action of NGF in brain injury:

- cholinergic neuroprotection: preservation of septohippocampal cholinergic neurons and neurotransmission [21,22];
- anti-inflammatory modulation: regulation of microglial and astrocytic phenotypes toward tissue repair [11];
- angiogenesis: stimulation of vascular growth and improved metabolic support to injured tissue [23,24].

**Figure 2:**
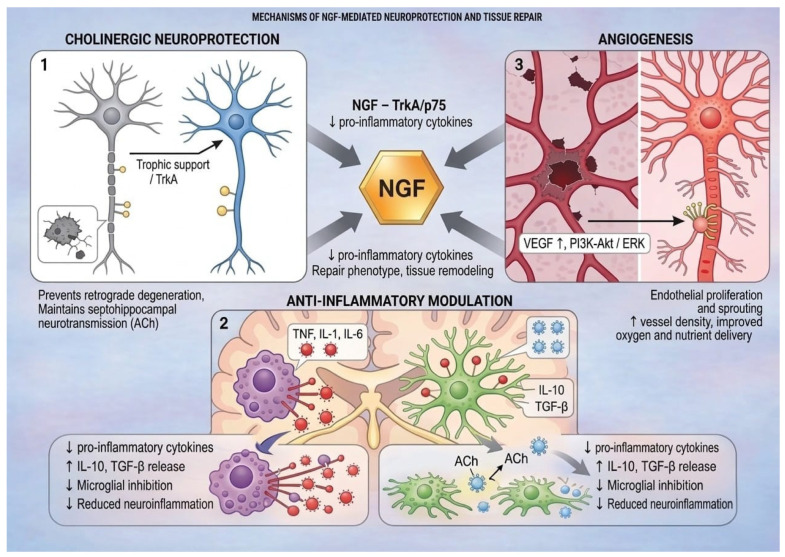
Multi-modal mechanisms of action of NGF in the injured brain.

Multi-modal mechanisms of action of NGF in the injured brain are shown in Figure 2.

### 3.2. Clinical Insights

Intranasal NGF administration enables the delivery of therapeutically relevant concentrations of the growth factor directly to the brain by bypassing the blood–brain barrier through the olfactory and trigeminal pathways, offering a safe, non-invasive, and well-tolerated approach. In this context, the pioneering work of Professor A. Chiaretti’s group includes clinical studies involving one child with meningitis, five patients with severe TBI, and three patients in a vegetative state after out-of-hospital cardiac arrest, providing the first human evidence for the feasibility and translational potential of intranasal NGF therapy [27,28,29,30,31].

#### 3.2.1. Use of Intranasal hr-NGF in a Patient with Neurological Impairment Following Late-Onset Meningitis

The first patient treated with intranasal NGF was a 7-week-old female infant with severe neurological sequelae following late-onset Streptococcus agalactiae meningitis [27]. Six months later, neurological evaluation showed profound disability, with a Level of Cognitive Functioning (LCF) score of 1, Disability Rating Scale (DRS) score of 26, and a Glasgow Outcome Scale (GOS) score of 2, with magnetic resonance imaging (MRI) evidence of multicystic encephalomalacia and tetraventricular hydrocephalus, and positron emission tomography (PET) findings of diffuse cerebral hypometabolism.

In the absence of alternative therapies, the patient received five monthly cycles of intranasal human recombinant NGF (hr-NGF, cenegermin, Oxervate^®^, Dompè Farmaceutici SpA, Milan, Italy) at 0.1 mg/kg, three times daily for 7 consecutive days. Neurological outcomes were monitored using serial clinical assessments and multimodal neuroimaging and neurophysiological studies.

**Figure 3:**
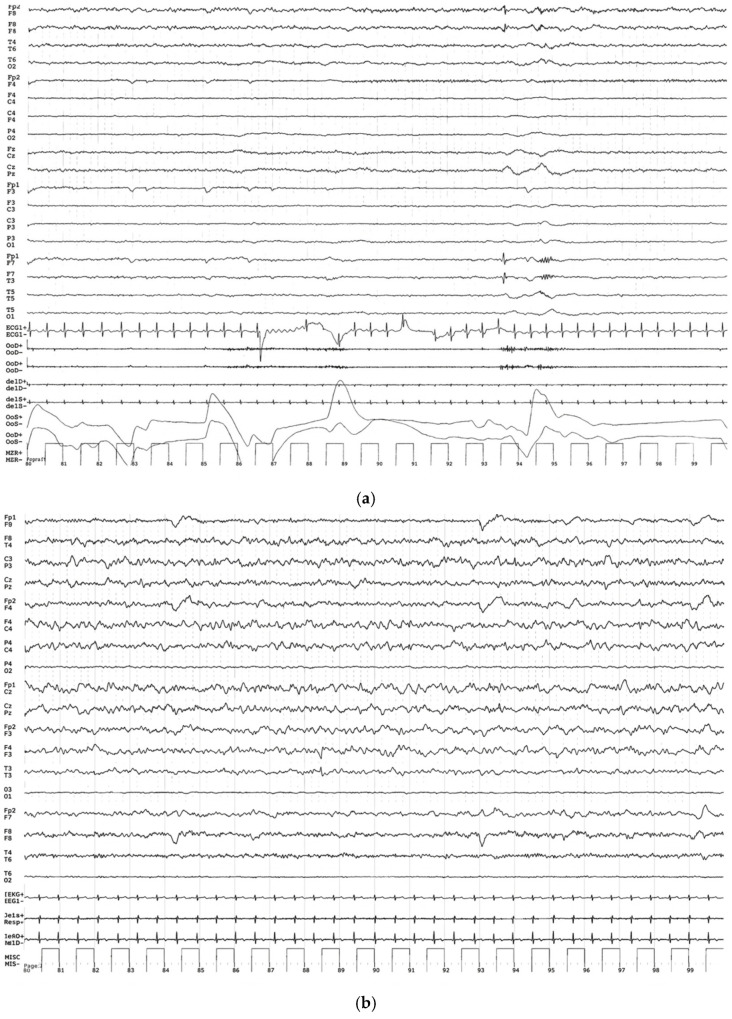
(**a**) EEG recordings obtained six months after the brain injury and prior to the start of NGF treatment revealed a severely depressed, low-voltage background activity, occasionally approaching an isoelectric pattern. Sporadic theta-delta discharges were observed in the right fronto-temporal regions. The distinction between wakefulness and sleep was poor, and overall cerebral activity appeared markedly reduced. (**b**) After completion of NGF treatment, the EEG displayed a clear improvement in cerebral electrical activity: a well-defined 4-5 Hz theta-delta background was evident bilaterally in the anterior regions (more prominent on the right), intermixed with abundant diffuse fast rhythms.

Following intranasal NGF treatment, functional neuroimaging showed objective improvements. PET demonstrated increased glucose metabolism in selected cortical and subcortical regions, supported by single-photon emission computed tomography (SPECT) evidence of enhanced cerebellar perfusion. EEG evolved from a near-isoelectric pattern to organized 4–5 Hz theta–delta background activity with diffuse fast rhythms (Figure 3a,b).

These instrumental changes were accompanied by clinically evident neurological recovery, including improved visual fixation and tracking, auditory recognition and orientation, normalization of ocular alignment, recovery of oral feeding, increased spontaneous and stimulus-related motor activity, restoration of corneal, vestibulo-ocular, and cough reflexes, partial recovery of hypothalamic functions, and improved outcome scores (LCF 2, DRS 21, GOS 3).

#### 3.2.2. Use of Intranasal hr-NGF in TBI Patients

Five pediatric patients with severe TBI and chronic disorders of consciousness were treated with intranasal hr-NGF [28]. All presented with prolonged coma, persistent motor and cognitive impairment, severe spasticity refractory to standard therapies, dysphagia requiring enteral feeding, and absent or severely impaired communication [28]. The treatment protocol consisted of intranasal hr-NGF Cenegermin (provided by Dompè Farmaceutici SpA, Milan, Italy) at 50 µg/kg/day, divided into three daily administrations for 7 days and repeated over four monthly cycles using a dedicated atomizer, after nasal cleansing [28].

Across the first three cases [28], intranasal hr-NGF was associated with improvements in consciousness, communication, oral feeding, motor control, and emotional expression. Patients showed recovery of facial expressivity, progressive resolution of oral-motor dyspraxia, partial restoration of swallowing, emergence of phonation, and improved voluntary movements. Spasticity was reduced, muscle tone and trophism improved, and autonomic functions such as cough reflex, thermoregulation, sleep–wake organization, and gastrointestinal motility were partially restored. Standardized scales documented objective gains, including increases in Gross Motor Function Measure (GMFM), reductions in Modified Ashworth Scale, and clinically meaningful improvements in the Disability Rating Scale (DRS), with all these patients transitioning from vegetative state or extreme vegetative state to severe disability classification [28].

**Figure 4:**
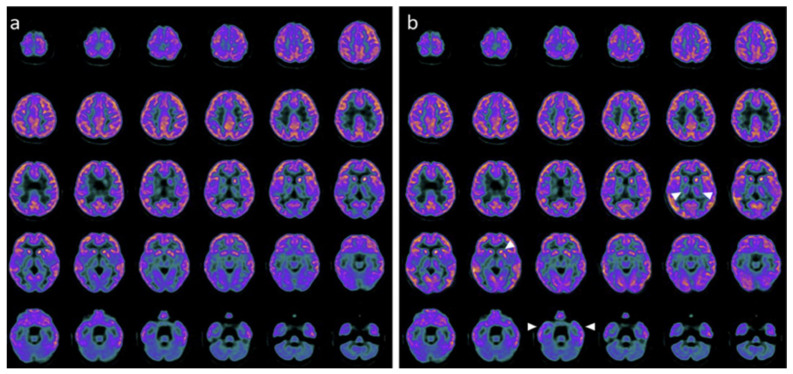
PET before and after the treatment with hr-NGF in the first TBI patient. (**a**,**b**) Brain 18F-FDG PET axial slices performed before (**a**) and after (**b**) intranasal hr-NGF treatment. A mild global reduction in 18F-FDG uptake was observed in all cortical regions, whereas a more marked reduction was detected in all subcortical regions (**a**). After hr-NGF administration, an increase in radiotracer uptake was found in the bilateral temporal cortex (right: +7%; left: +7%), right and left thalamus (+6% and +4%, respectively), and the left caudate nucleus (+9%). The white arrows delineate the aforementioned areas. (**b**).

**Figure 5:**
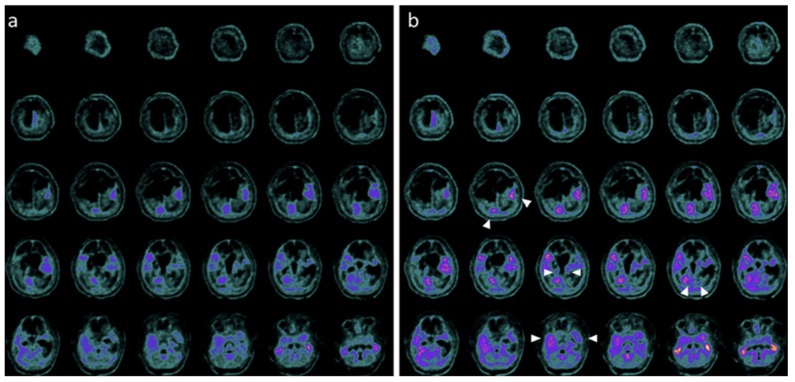
PET before and after the treatment with hr-NGF in the second TBI patient. (**a**,**b**) Brain 18F-FDG PET axial slices performed before (**a**) and after (**b**) hr-NGF treatment. A severe global reduction in 18F-FDG uptake was observed in all cortical and subcortical regions as well as in the cerebellum (**a**). After hr-NGF administration, an increase in radiotracer uptake was detected in the left and right temporal cortex (right: +18%; left: +15%), bilateral parietal cortex (right: +18%; left: +15%), right and left occipital cortex (right: +10%; left: +13%), and right and left thalamus (+7% and +8%, respectively). The white arrows delineate the aforementioned areas. (**b**).

**Figure 6:**
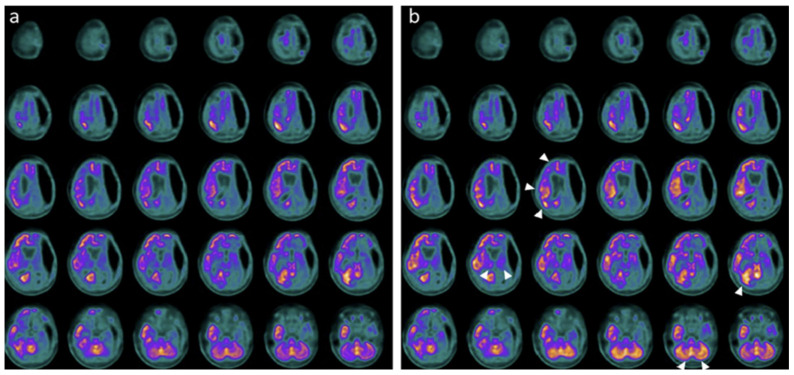
PET before and after the treatment with hr-NGF in the third TBI patient. (**a**,**b**) Brain 18F-FDG PET axial slices performed before (**a**) and after (**b**) hr-NGF treatment. A severe reduction in 18F-FDG uptake was observed in all cortical and subcortical regions of the left hemisphere, whereas a mild reduction was detected in the whole cerebellum and all cortical and subcortical regions of the right hemisphere (**a**). After hr-NGF administration, an increase in radiotracer uptake was found in the right frontal cortex (+11%), right temporal cortex (+15%), right parietal cortex (+14%), right occipital cortex (+22%), right and left thalamus (+10% and +7%, respectively), and cerebellum (+33%). The white arrows delineate the aforementioned areas. (**b**).

Neuroimaging and neurophysiology supported these clinical changes. Baseline PET and SPECT showed markedly reduced tracer uptake across cortical, subcortical, thalamic, and cerebellar regions, while post-treatment studies demonstrated regional metabolic and perfusion increases, particularly in the temporal, parietal, occipital cortices, thalami, and cerebellum (Figure 4, Figure 5 and Figure 6) [28].

EEG recordings evolved from diffuse low-voltage, poorly organized activity to more structured rhythms with reduction in slow frequencies and relative increase in faster components, as confirmed by power spectral density (PSD) analysis [28]. No significant adverse events were reported, and treatment was well-tolerated with excellent family compliance [28].

In the fourth case [29], a 3-year-old boy with severe TBI showed major motor and functional recovery after intranasal hr-NGF, achieving independent balance, autonomous ambulation, and intentional use of the previously paretic upper limb. Cognitive testing revealed measurable improvements in visuospatial reasoning, working memory, processing speed, and expressive language. EEG showed a reduction in epileptiform activity, and MRI confirmed radiological stability without new lesions [29].

**Figure 7:**
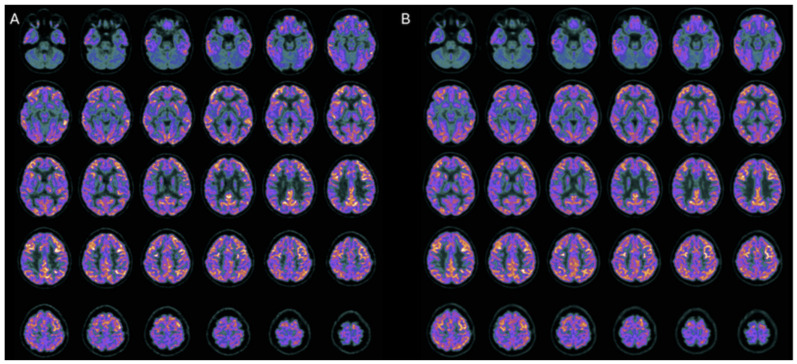
18F-FDG PET brain axial slices performed before (**A**) and after (**B**) NGF treatment in the fifth TBI patient. Mild reduction in 18F-FDG uptake in the bilateral parietal cortex, subcortical regions, and a slightly more severe reduction in cerebellum (**A**). After the NGF administration, an increase of the radiotracer uptake in the bilateral parietal cortex (right: +7%; left: +6%), right and left caudate nucleus (right: +3%; left: +5%), and cerebellum (+6%) was observed (**B**).

In the fifth case [30], a 14-year-old boy with severe diffuse axonal injury exhibited significant cognitive recovery after intranasal hr-NGF, with improvements in overall intellectual functioning, processing speed, working memory, attention, inhibitory control, visuospatial memory, academic skills, and adaptive behavior [30]. PET showed increased glucose metabolism in the parietal, striatal, and cerebellar regions (Figure 7), while SPECT demonstrated improved perfusion in the caudate nuclei, putamen, and thalami [30]. EEG remained physiologically normal throughout treatment [30].

Overall, these clinical cases indicate that intranasal hr-NGF is safe, feasible, and associated with coherent improvements across the clinical, neuropsychological, neuroimaging, and electrophysiological domains in severe pediatric TBI [28,29,30]. The convergence of behavioral recovery with objective biomarkers strongly supports a biologically interesting effect rather than nonspecific rehabilitation-related changes.

#### 3.2.3. Use of Intranasal hr-NGF in HIE Patients

Three children with chronic vegetative state secondary to out-of-hospital cardiac arrest were treated with intranasal hr-NGF [31]. Two were male, and the mean age at evaluation was 36 months [31]. All showed severe neurological impairment, including disorders of consciousness, spasticity, respiratory failure requiring tracheostomy and assisted ventilation, and enteral feeding dependence [31]. Baseline PET and SPECT revealed widespread reductions in cerebral metabolism and perfusion, particularly involving the thalami, brainstem, striatum, cerebellum, and mesial temporal regions, with variable cortical involvement across patients [31].

Treatment consisted of intranasal cenegermin-bkbj (rh-NGF) ophthalmic solution 0.002% (20 mcg/mL) (Oxervate, Dompè Farmaceutici SpA, Milan, Italy), at 50 µg/kg, administered three times daily for 10 days and repeated twice at one-month intervals, combined with anodal transcranial direct current stimulation (tDCS) over the left dorsolateral prefrontal cortex using a constant-current stimulator (NeuroConn, DC Stimulator MC) and saline-soaked sponge electrodes, for 10 consecutive days per cycle [31]. tDCS was delivered at 2 mA for 20 min and then reduced back to 0 over 5 s at the end of the session, using a standardized protocol [31].

**Figure 8:**
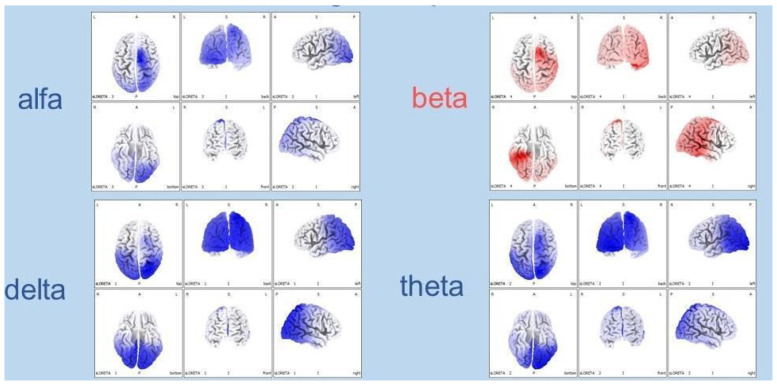
In the three HIE patients, the topographical analysis of the post-treatment EEG PSD distribution revealed a reduction in slow-frequency delta and theta bands—typically predominant in severe brain dysfunction—a milder decrease in the alpha band, and an increase in fast beta activity, usually associated with active thinking, concentration, and alert wakefulness. The observed spectral power shift from low-frequency (slow) to high-frequency (fast) bands may represent a neurophysiological marker of restored cerebral reactivity and a potential sign of clinical improvement.

Following combined hr-NGF and tDCS therapy, all patients showed improvements in functional neuroimaging and electrophysiological measures, with PET and SPECT demonstrating increased metabolic activity and perfusion in previously hypoactive regions, and EEG/PSD analyses indicating more organized cerebral electrical activity (Figure 8) [31].

**Figure 9:**
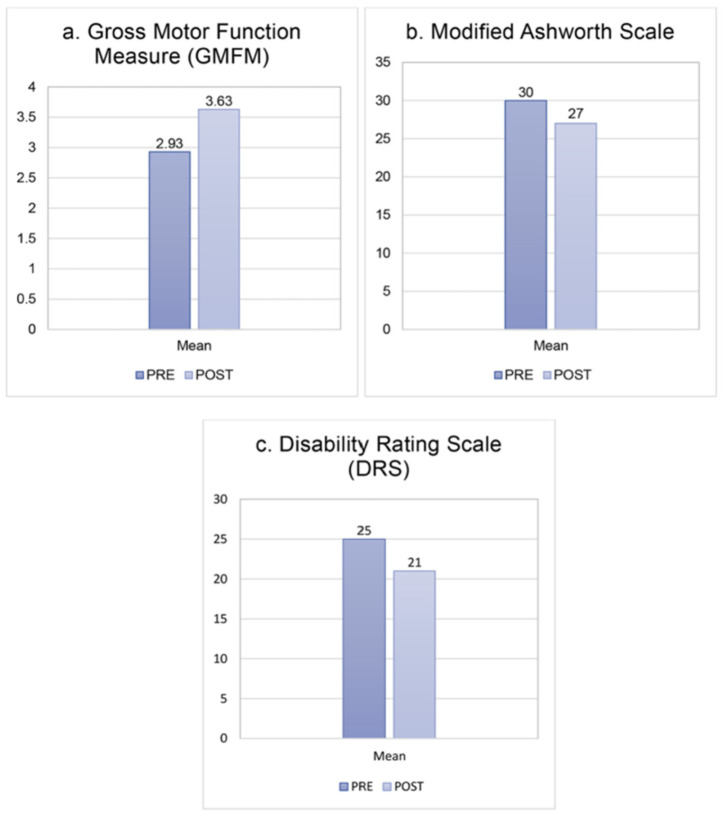
Changes in the clinical scales used to detect the improvement of HIE children after the treatment with hr-NGF and tDCS. (**a**) A mean improvement of 23% of GMFM (from 2.93to3.63%) was recorded after the treatment. (**b**) A mean improvement of 3 points on the modified Ashworth Scale showed a post treatment reduction in hypertonus. (**c**) An improvement of 4 points in DRS was observed in all treated patients.

Clinically, patients exhibited reduced spasticity, emergence of voluntary finger movements, and enhanced facial expressiveness (Figure 9). No adverse effects were reported [31].

Overall, this case series suggests that intranasal hr-NGF, especially when combined with neuromodulation, may promote recovery not only in motor function, but also in the cognitive and relational domains, including executive functions, impulse control, and environmental awareness, with a meaningful impact on quality of life and social interaction [31].

All analyzed cases are resumed in Table 1.

## 4. Discussion

Intranasal administration of hr-NGF emerged from this review as a biologically plausible and clinically promising strategy to enhance neurorepair in children with acquired brain injury, although current evidence is still preliminary and largely based on small, uncontrolled studies. The converging preclinical and clinical data suggest that hr-NGF can modulate multiple pathophysiological pathways implicated in traumatic and hypoxic-ischemic damage, thereby promoting functional recovery beyond spontaneous or rehabilitation-driven improvement [2].

In experimental models of TBI and HIE, NGF acts as a key component of the endogenous response to injury, with astrocytic upregulation reflecting an intrinsic attempt to counteract excitotoxicity, neuroinflammation, and apoptosis [8,22]. Exogenous NGF administration, including via intranasal delivery, reduces lesion size, preserves vulnerable neuronal populations, supports oligodendrocyte survival and remyelination, and enhances angiogenesis through VEGF-related pathways and activation of TrkA–PI3K/Akt–ERK1/2 signaling [23]. These multimodal effects translate into improved motor and cognitive performance in animal models, strengthening the rationale for clinical translation in pediatric populations [26]. Of particular interest, the combination of NGF with therapeutic hypothermia or other neuroprotective interventions appears synergistic in HIE models, indicating that hr-NGF may best be conceived as part of a broader multimodal strategy rather than a stand-alone therapy [26].

The clinical literature, although limited to a single meningitis case, five TBI cases, and three HIE patients, provides coherent evidence that intranasal hr-NGF can reach the developing brain and induce measurable changes across the behavioral, neuroimaging, and electrophysiological domains [27,28,29,30,31]. In severe TBI, intranasal hr-NGF was associated with the recovery of consciousness, reduction in spasticity, improved voluntary motor control, and gains in communication and higher-order cognitive functions, paralleled by increased cortical and subcortical metabolism on PET/SPECT and normalization of EEG patterns [28,29,30]. Similarly, in HIE patients with chronic disorders of consciousness, combined hr-NGF and tDCS led to reduced spasticity, emergence of voluntary movements, and enhanced relational and executive abilities, again supported by improved cerebral perfusion and more organized EEG activity [31]. Notably, across all reports intranasal hr-NGF was well-tolerated, with no treatment-related serious adverse events and excellent family compliance, underscoring the feasibility and safety of this non-invasive route in fragile pediatric patients.

Nonetheless, several limitations temper the strength of these findings and must be acknowledged to avoid overinterpretation. First, all clinical data derived from case reports and small case series without control groups, randomization, or blinding, which makes it impossible to disentangle the specific contribution of hr-NGF from spontaneous recovery, rehabilitative interventions, and placebo effects. Since our research group has been the primary contributor to this specific field of study to date, the available clinical evidence originates from a single investigative program; consequently, independent replication is currently lacking, and these data represent the only evidence available to guide current clinical understanding. A substantial proportion of the cited clinical evidence originates from the authors’ research group, reflecting the limited number of independent studies currently available in this field. This limitation should be considered when interpreting the findings. Critically, reliance on data generated exclusively by a single investigative program introduces a significant risk of systematic error, as any inherent methodological biases may have been propagated across all included studies. The heterogeneity of etiologies, ages at injury and treatment, lesion topography, concomitant therapies, and follow-up durations further complicates cross-case comparisons and precludes robust conclusions on optimal timing, dosing, or patient selection. In HIE, the concurrent use of tDCS introduces an additional confounder, as the relative weight of neuromodulation and hr-NGF cannot be isolated from the combined protocol. Moreover, in order to draw clear conclusions, it is also necessary to consider the difficulty of distinguishing the contribution of hr-NGF from the effects of neuromodulation and rehabilitation. Furthermore, outcomes were assessed using different clinical scales and neuropsychological tools across studies, limiting standardization and the possibility of quantitative synthesis. From a mechanistic perspective, although neuroimaging and EEG findings are consistent with the reactivation of hypoactive networks, direct biomarkers of NGF bioavailability, target engagement, and downstream pathway activation in humans are still lacking. Another limitation of this review lies in the substantial heterogeneity of the included studies, which encompass preclinical models, retrospective series, and prospective clinical trials. This inclusive approach was necessitated by the marked paucity of high-level evidence currently available in the literature regarding intranasal NGF in pediatric populations. While the integration of diverse study designs introduces inherent methodological bias and complicates direct comparative analysis, it was essential to provide a comprehensive state-of-the-art synthesis of this nascent field.

These gaps delineate clear priorities for future research. As the clinical data were derived from small, uncontrolled case series, our statements should currently be considered hypothesis-generating. Notably, various clinical trials investigating the intranasal administration of cenegermin are currently in progress. Large, multicenter, randomized controlled trials are urgently needed to confirm the safety and efficacy of intranasal hr-NGF in well-defined pediatric TBI and HIE populations, with standardized protocols for dosing, timing, and duration of treatment. Stratification by age, severity, lesion pattern, and comorbidities will be essential to identify the subgroups most likely to benefit and to understand whether hr-NGF is more effective in the subacute or chronic phases of injury. Trials should incorporate harmonized outcome measures, including validated clinical scales, quantitative neuropsychological assessments, and multimodal biomarkers (advanced MRI, PET/SPECT, EEG/PSD, and fluid biomarkers of neuroaxonal injury and inflammation) to capture both the functional and mechanistic effects. In parallel, translational studies are needed to refine intranasal formulations and delivery systems, characterize pharmacokinetics and pharmacodynamics in children, and explore rational combinations with established and emerging neuroprotective or neuromodulatory interventions.

## 5. Conclusions

Preclinical evidence consistently shows that NGF is a key component of the brain endogenous response to injury and a strong therapeutic candidate. Its upregulation by astrocytes after trauma or hypoxia reflects a natural protective mechanism that can be pharmacologically enhanced. Intranasal NGF administration rescues vulnerable neurons, reduces neuroinflammation, improves tissue perfusion, and promotes cognitive and motor recovery in animal models.

Available clinical data, although still limited, indicate that intranasal hr-NGF can reactivate neurobiological circuits in the developing brain and promote neurorepair and functional plasticity even months after injury. Measurable improvements in both motor outcomes and higher-order cognitive functions suggest a genuine disease-modifying potential rather than a purely symptomatic effect. This approach therefore represents a promising new frontier for severe pediatric neurological sequelae.

However, current evidence still remains preliminary. Large, well-designed, multicenter, randomized, long-term controlled trials are essential to confirm safety and efficacy, optimize dosing and timing, and define which patient subgroups are most likely to benefit from this innovative therapeutic strategy.

## Figures and Tables

**Table 1:** An overview of currently available clinical studies on intranasal hr-NGF.

Population	Intervention	Key Findings	Concomitant Interventions	Reference
A 7-month-old girl with neurological impairment following late-onset meningitis	0.1 mg/kg of hr-NGF, administered intranasally, three times a day for 7 consecutive days for 5 cycles, at one month distance each	NGF therapy improved selected cortical and subcortical regions metabolism, cerebellar perfusion, cerebral electric activity, visual fixation and tracking, auditory recognition and orientation, ocular alignment, oral feeding, motor activity, corneal, vestibulo-ocular, and cough reflexes, and hypothalamic functions	None	Chiaretti et al., 2020 [27]
3 children (mean age 36 months) affected by chronic vegetative state secondary to out-of-hospital cardiac arrest	50 µg/kg of hr-NGF, administered intranasally, combined with tDCS three times a day for 10 consecutive days for 2 cycles, at one month distance each	Combined NGF and tDCS therapy improved functional neuroimaging and electrophysiological measures, with increased metabolic activity and perfusion in previously hypoactive regions, while it reduced spasticity, and enhanced facial expressiveness	tDCS	Curatola et al., 2023 [31]
3 children aged 3 to 10 years with severe TBI sequelae	50 µg/kg of hr-NGF, administered intranasally, three times a day for 7 consecutive days for 4 cycles, at one month distance each	NGF therapy reduced spasticity and improved the recovery of facial mimicry, voluntary movements, oral motor function, verbal comprehension, attention, cough reflex, crying ability, and feeding abilities	None	Gatto et al., 2023 [28]
A 14-year-old boy with severe TBI sequelae	50 µg/kg of hr-NGF, administered intranasally, three times a day for seven consecutive days for 4 cycles, at one month distance each	NGF administration improved radiological functional pattern, cognitive processes, memory, communication strategy, execution skills, attention and verbal expression	None	Capossela et al., 2024 [30]
A 3-year-old boy with severe TBI sequelae	50 µg/kg of hr-NGF, administered intranasally, three times a day for seven consecutive days for 4 cycles, at one month distance each	NGF therapy improved motor function, verbal comprehension, executive functions and EEG pattern	Rehabilitation	Di Sarno et al., 2025 [29]

## Data Availability

No new data were created or analyzed in this study. Data sharing is not applicable.

## Abbreviations

The following abbreviations are used in this manuscript:

18F FDG	Fluorine 18 fluorodeoxyglucose
Akt	Protein kinase B
CENTRAL	Cochrane Central Register of Controlled Trials
CCI	Controlled cortical impact
CNS	Central nervous system
CRS-R	Coma Recovery Scale–Revised
DRS	Disability Rating Scale.
EEG	Electroencephalography
ERK1/2	Extracellular signal-regulated kinases 1 and 2
FPI	Fluid percussion injury
GBS	Group B Streptococcus
GCS	Glasgow Coma Scale
GMFM	Gross Motor Function Measure
GOS	Glasgow Outcome Scale
HIE	Hypoxic-ischemic encephalopathy
HIF-1α	Hypoxia-inducible factor 1-alpha
hr-NGF	Human recombinant nerve growth factor
LCF	Level of Cognitive Functioning
MRI	Magnetic resonance imaging
NGF	Nerve growth factor
PET	Positron emission tomography
PI3K	Phosphoinositide 3-kinase
PRISMA	Preferred Reporting Items for Systematic Reviews and Meta-Analyses
PSD	Power spectral density
SPECT	Single-photon emission computed tomography
tDCS	Transcranial direct current stimulation
TBI	Traumatic brain injury
TrkA	Tropomyosin receptor kinase A
VEGF	Vascular endothelial growth factor
